# Dry eye in Parkinson's disease: a narrative review

**DOI:** 10.3389/fneur.2023.1236366

**Published:** 2023-08-01

**Authors:** Larisa Ungureanu, K. Ray Chaudhuri, Stefania Diaconu, Cristian Falup-Pecurariu

**Affiliations:** ^1^Department of Neurology, County Clinic Hospital, Braşov, Romania; ^2^Faculty of Medicine, Transilvania University, Braşov, Romania; ^3^Department Basic and Clinical Neuroscience, Parkinson Foundation Centre of Excellence, King's College London, Denmark Hill Campus, King's College Hospital, The Maurice Wohl Clinical Neuroscience Institute, London, United Kingdom

**Keywords:** dry eye, Parkinson's disease, keratoconjunctivitis sicca, visual disturbances, diagnosis, management

## Abstract

In Parkinson's disease (PD) patients, a wide range of ocular and visual disorders are present. Tear film instability, inflammation and dysfunction of the ocular surface, and the presence of symptoms of visual disturbance characterize dry eye, a multifactorial disease of the ocular surface. Based on a literature search, we discuss the frequency, pathogenesis, and influence on the quality of life of patients with dry eye in Parkinson's disease. Furthermore, we review the available means of diagnosis and management of dry eye. An improvement in awareness and recognition of dry eye is needed to provide suitable, personalized therapeutic options for PD patients, aiming to improve their quality of life, independence, and safety.

## Introduction

Parkinson's disease (PD) is a neurodegenerative disorder presenting a wide range of non-motor symptoms including visual disturbances, with important implications for the quality of life of these patients. Visual disturbances in PD range from peripheral to central and include dry eye, diplopia, decreased blink rates, blepharitis, blepharospasm, visual hallucinations, retinal abnormalities, and convergence insufficiency (1). These disturbances lead to the appearance of ocular symptoms such as eye tearing, blurred vision, difficulty with reading, doubling of images, presence of passage hallucinations, impaired contrast sensitivity, and color vision and are frequently interconnected. For example, dopamine depletion and alpha-synuclein aggregation in the cell layers of the intra-retinal region have been shown to lead to a dysfunction in visual processing with impairment in color discrimination, contrast sensitivity, visual acuity, object, and motion perception (2, 3); double vision has been associated with the presence of visual hallucinations and convergence insufficiency (4). Dry eye disease was defined by the 2007 International Dry Eye Workshop (5) as “a multifactorial disease of the tears and ocular surface that results in symptoms of discomfort, visual disturbance, and tear film instability with potential damage to the ocular surface that is accompanied by increased osmolarity of the tear film and inflammation of the ocular surface”. Symptoms of dry eyes include excess tearing, stinging or burning eyes, foreign body sensation, scratchiness, photophobia, and redness of the eye (6).

Dry eye disease has a prevalence of as high as 70% in PD patients (1, 7). Thus, patients with PD should be considered to be at increased risk of developing dry eyes. The aim of this study was to review the pathogenesis, clinical evaluation, impact on quality of life (QoL), and management of dry eye in Parkinson's disease.

## Pathogenesis of dry eye in Parkinson's disease

**Figure 1:**
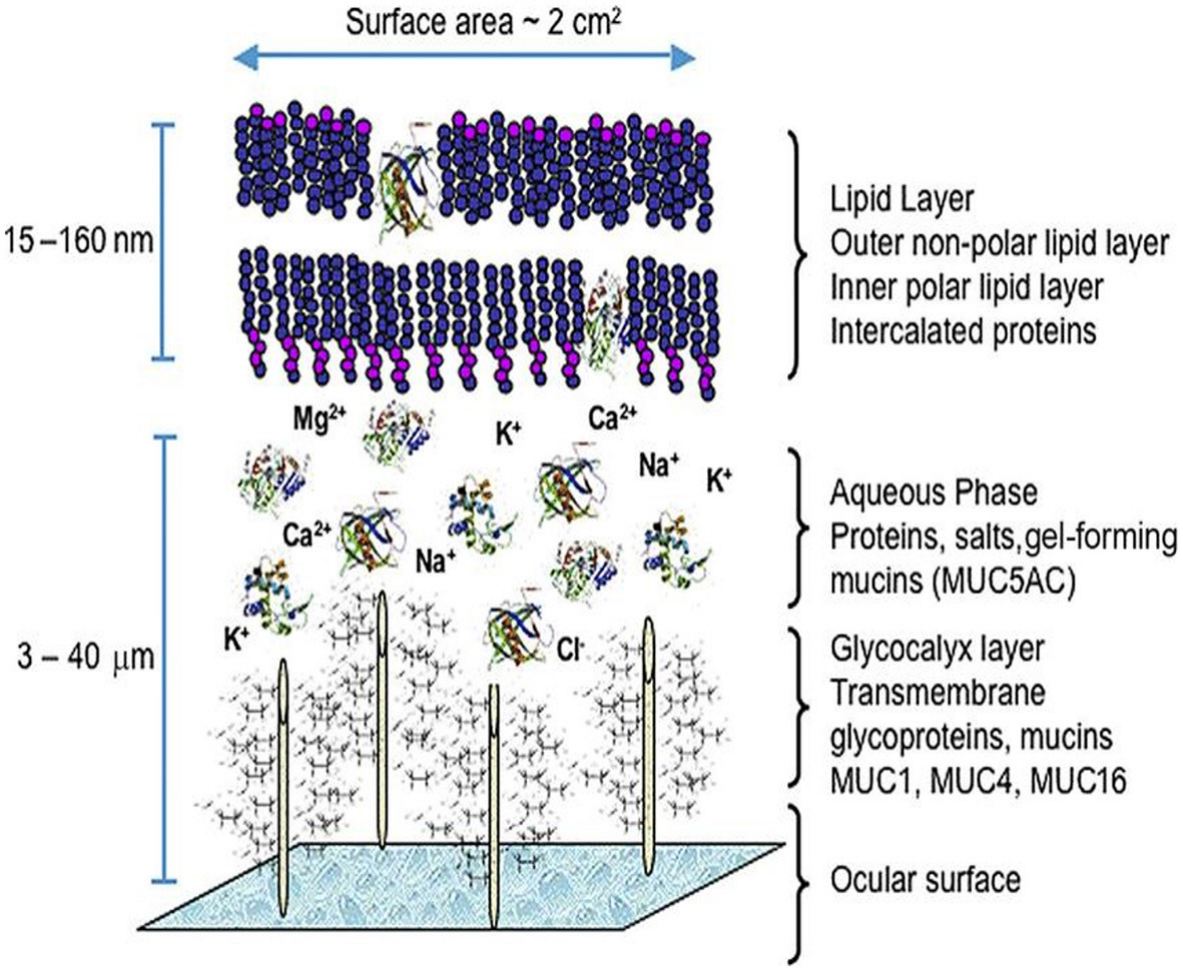
The precorneal tear film model. From Green-Church et al. (90), with permission.

Dysfunction of the tear-secreting glands and/or disorders of the ocular surface led to dry eye (8). The precorneal tear film is a hydrated gel, with its composition including water, electrolytes, mucins, soluble antimicrobial proteins (lactoferrin and lysosome), immunoglobulins, and growth factors that help regulate cellular processes (9, 10). A superficial lipid layer formed by hydrophilic polar lipids such as phospholipids and ceramides is adjacent to the aqueous-mucin layer (11). The aqueous-mucin layer is anchored by chemical attractions to the superficial corneal epithelium (Figure 1) (12). Its role is to protect and support the ocular surface.

The communication between the ocular surface (cornea and conjunctiva) and the tear-secreting glands (lacrimal glands and meibomian glands) occurs through a neural reflex arc: The sensory afferent information travels through the ophthalmic branch of the fifth cranial nerve (the trigeminal nerve) to the pons where the integration of signals with the input from cortical and other central nervous system centers is made. The efferent nerves are both parasympathetic (seventh cranial nerve—the facial nerve) and sympathetic (paraspinal sympathetic chain) fibers that travel to the lacrimal glands and are responsible for tear secretion (9). A decrease in aqueous tear secretion leads to an increase in tear film osmolarity and chronic inflammation that may severely affect the function and differentiation of the ocular surface epithelium (13).

**Figure 2:**
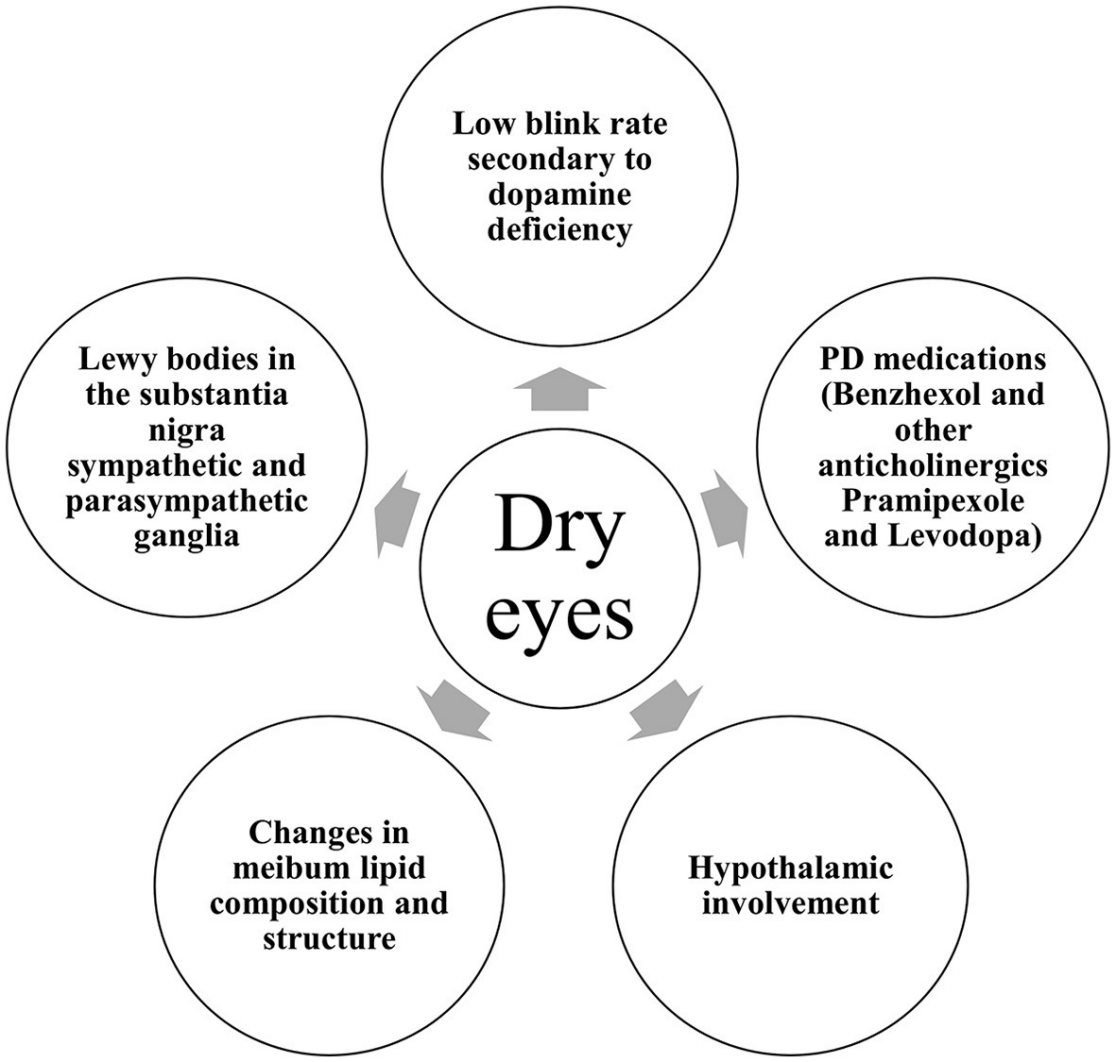
Mechanisms of dry eye in Parkinson's disease.

In Parkinson's disease, several mechanisms are incriminated (Figure 2). With disease progression, the accumulation of aggregated alpha-synuclein was hypothesized to spread from the nigrostriatal dopaminergic system to the hypothalamus and neocortex in a caudo-rostral pattern, leading to cell dysfunction, degeneration, and a subsequent decrease in striatal dopamine levels (14). The neurochemical control of blinking is exerted by the dopaminergic, GABAergic, and cholinergic systems of the brainstem. Thus, the decreased levels of dopamine in the central nervous system (CNS) of PD patients give rise to significantly decreased blink rates (15). Blinking is crucial for maintaining an adequate tear film on the surface of the eyes. Second, it is acknowledged that abnormalities in autonomic function are ubiquitous in PD (16). The superior salivatory nucleus and the lacrimal nucleus in the pons give rise to general visceral (parasympathetic and sensory) efferent fibers that are carried to the geniculate ganglion within the intermediate nerve. Preganglionic parasympathetic fibers exit the geniculate ganglion forming the greater superficial petrosal nerve that joins the deep petrosal nerve toward the pterygopalatine ganglion. From there, parasympathetic postganglionic fibers synapse with the lacrimal glands (17). From the hypothalamus, descending autonomic fibers regulated by the ventral striatum and limbic system travel to the superior salivatory nucleus. Thus, the dysfunction of the autonomic system caused by the presence of Lewy bodies in the substantia nigra as well as the sympathetic and parasympathetic ganglia might explain the lacrimation disturbances found in PD patients (18). Furthermore, changes in meibum lipid composition and structure could contribute to the increased susceptibility to dry eye in PD patients (19). Tear proteins involved in lipid metabolism, oxidative stress, and immune response were found to be altered in Parkinson's disease patients (20).

Finally, antiparkinsonian medications were associated with dry eye syndrome (21). Benzhexol, pramipexole, and levodopa are known to cause dryness in mucous membranes due to their anticholinergic effects. Other examples include orphenadrine, benztropine, bornaprine, procyclidine, benapryzine, and methixine (22).

## Clinical evaluation of dry eye

### Subjective assessment

Dry eye can be assessed by using rating scales and questionnaires. The description, scoring system, and validity parameters of questionnaires can be found in Table 1. The Ocular Surface Disease Index (OSDI) is the most commonly used dry eye questionnaire. In PD patients, OSDI scores were significantly higher compared to healthy subjects (7).

**Table 1:** Description, scoring system, and validity parameters of available dry eye questionnaires.

**Questionnaire**	**Description**	**Scoring**	**Validity parameters**
Ocular surface disease index (OSDI)	The most used one. 12-item questionnaire; assessment of symptoms of ocular irritation and their impact on vision-related functioning. Subscales: vision-related function, ocular symptoms, and environmental triggers.	Total score of 0 to 100 Higher score = greater disability 0–12 = normal; 13–22 = mild dry eye; 23–32 = moderate dry eye; >33 = severe dry eye.	*Sensitivity* 0.60–0.92(higher in more severe diseases) *Specificity* 0.83 *Reliability* Internal consistency (Cronbach's α = 0.92 (0.84–0.94) Area under curve ROC = 0.970 Schiffman et al. (81)
Visual Functioning Questionnaire-25 (NEI-VFQ-25)	25-item questionnaire; shorter version of NEI-VFQ; assessment of the effect of visual impairment on the patient's health-related quality of life	Total score of 0 to 100 Higher score = greater disability	Reliability^*^ Internal consistency = between 0.66 and 0.94 for all subscales ^*^for NEI-VFQ, longer 51-item-questionnaire Mangione et al. (82)
Standardized Patient Evaluation of Eye Dryness (SPEED) Questionnaire	Eight items; assessment of frequency and severity of symptoms, monitoring of diurnal and changes in symptoms over 3 months	Total score of 0–28 0–4 = mild dry eye; 5–7 = moderate dry eye; >8 = severe dry eye.	*Reliability* Internal consistency = between 0.86 and 0.95 Area under curve ROC = 0.928 Ngoet al. (83)
Dry Eye Questionnaire 5 (DEQ-5)	5-item questionnaire; assessment of the frequency of watery eye, discomfort and dryness, and late-day discomfort and dryness intensity; simplified version of the original DEQ	Total score of 0–22 >6 = dry eye; >12 = suspected Sjogren syndrome (SS).	*Sensitivity* 0.712 *Specificity* 0.827 *Reliability* Internal consistency = 0.819 Area under curve ROC = 0.835 Akowuah et al. (84)
Symptom Assessment in Dry Eyes (SANDE) Questionnaire	Two questions presented in a visual analog scale; assesses the frequency and severity of dry eye syndrome, and is useful in detecting changes in symptoms over time.	Visual analog scale Frequency of symptoms: rarely to all the time Severity of symptoms: very mild to very severe	*Reliability* Spearman coefficient correlation*: R* = 0.64; *P* < 0.001 (when compared to OSDI) Amparo et al. (85) *Repeatability* Intraclass correlation coefficient (ICC) = 0.53–0.76 Schaumberg et al. (86)
Dry Eye related Quality of life Score (DEQS)	15-item questionnaire; assessment of dry eye symptoms and influence on daily life, and the overall degree of quality of life impairment	Total score of 0–100 Higher score = greater disability	*Reliability* Internal consistency (Cronbach's α) = 0.93 *Repeatability* Intraclass correlation coefficient (ICC) = 0.91 Sakane et al. (87)
McMonnies Questionnaire	14-item questionnaire; helps detect dry eye, detects patients at risk for developing dry eye due to exposure to specific factors	Specific scoring systems available	*Sensitivity* 0.87–0.93 *Specificity* 0.85–0.89 *Reliability* Area under curve ROC = 0.94 Gothwal et al. (88)
The University of North Carolina Dry Eye Management Scale (UNC DEMS)	Single-item scale; provides a snapshot of a patient's overall experience: symptoms and quality of life over the last week	Visual analog scale From 1 (no symptoms) to 10 (severe symptoms)	*Reliability* Spearman coefficient correlation: *R* = 0.80; *P* < 0.001 (when compared to OSDI) *Repeatability* Test–retest reliability coefficient = 0.90 Grubbs et al. (89)

### Objective assessment

Tear function abnormalities in PD patients have been reported in the literature. Most authors reported more than one abnormal tear film test result, and, in some cases, the results were correlated with disease severity.

#### Slit lamp examination

A slit lamp examination can be used to diagnose moderate-to-severe dry eye by measuring the upper and lower tear menisci and by assessing the presence and grade of lid-parallel conjunctival folds (LIPCOFs), a sensitive predictor of dry eye. Based on the examination of conjunctival folds in the lower temporal quadrant, a grading system of three degrees has been proposed (23). The LIPCOF degree did not significantly differ between patients with Parkinson's disease and controls in a study by Nowacka et al. (7).

Therefore, more studies are needed in order to assess the usefulness of slit lamp examination for dry eye disease in PD.

#### Aqueous tear production (Schirmer test)

The insertion of a standardized filter paper strip into the lower conjunctival sac in order to measure the amount of wetting (millimeter units) after a period of 5 min is known as the Schirmer test. The screening threshold for dry eyes is 10 mm, with a value of 5 mm or less confirming the diagnosis (6).

Schirmer test scores were significantly lower in patients with Parkinson's disease than those in controls in a study by Demirci et al. (24) (6.52 ± 2.94 mm/5 min vs. 11.3 ± 6.16 mm/5 min). Similar results were obtained in three other studies (25–27) that followed the corneal parameters in PD patients compared to controls: 6.56 ± 4.75 mm/5 min vs. 12.81 ± 5.68 mm/5 min, 9.08 ± 4.46 mm/5 min vs. 17.16 ± 9.57 mm/5 min, and 4.3 ± 1.8 mm/5 min vs. 9.4 ± 3.0 mm/5 min, respectively. Schirmer's test scores were also found to be significantly affected in patients with PD compared to healthy subjects (13.20 ± 10.45 vs. 17.49 ± 11.16 mm) in a study by Nowacka et al. (7).

The Schirmer test, therefore, is a useful method for evaluating and diagnosing dry eye in Parkinson's disease patients.

#### Staining of the ocular surface

Vital staining of the ocular surface using different dyes, such as lissamine green, rose bengal, and fluorescein, has been widely used to assess the integrity of the conjunctival and corneal epithelial cells. The damaged epithelial cell stain was in a bright color (green for fluorescein and lissamine green and purple for rose bengal) after the instillation of a drop of dye solution under cobalt-blue-filtered light (28).

Reddy et al. (29) determined the degree of ocular surface staining with rose bengal, lissamine green, and fluorescein sodium in patients with PD and progressive supranuclear palsy (PSP) compared to healthy subjects. A high percentage of PD and PSP patients had abnormal staining compared to healthy controls (none). This is in concordance with a study by Demirci et al. (24) that found higher corneal fluorescein staining in PD patients than that in the control group.

Staining of the ocular surface may prove useful in diagnosing dry eye disease in PD, but more studies are needed.

#### Tear film stability (tear break-up time)

By applying a fluorescein strip to the lower conjunctival sac and examining it under cobalt-blue-filtered light, the tear break-up time or TBUT can be determined (30). TBUT represents the time measured between the last blink and the appearance of the first dark spot, and a value under 10 s is considered abnormal (31).

TBUT was significantly lower in patients with Parkinson's disease than in healthy subjects in various studies (18, 24, 25, 32). Biousse et al. (1) found that only TBUT was abnormal in PD patients compared to controls in terms of normal rose bengal staining and Schirmer test values. In another study, TBUT was not significantly different between the PD group and the control group, while Schirmer test results and meibomian gland function were significantly affected (7).

Studies are conflicting regarding the use of TBUT in properly diagnosing dry eye syndrome. More studies are needed in order to assess the usefulness of the test for Parkinson's disease patients.

#### Anterior segment optical coherence tomography

With high-resolution AS-OCT, cross-sectional images of the cornea can be obtained, allowing not only the examination of corneal layers (38) but also the measurement of corneal thickness and cross-sectional area, precise height, and volume of the tear meniscus (33).

Using AS-OCT, Ulusoy et al. (25) measured the thickness of each corneal sublayer in patients with Parkinson's disease in comparison to healthy individuals. They found that the thicknesses of the Bowman and stromal layers were significantly lower in PD patients. Furthermore, stromal thickness was negatively correlated with disease duration and severity and positively correlated with TBUT and Schirmer test scores. They concluded that reduced blinking rates and tear film dysfunction lead to corneal thinning in patients with PD.

Corneal thickness is an important indicator of corneal health. Central corneal thickness (CCT) was found to be significantly decreased in PD patients compared to healthy subjects in several studies (24).

Aksoy et al. (32) reported that the CCT, TBUT, and Schirmer test values decrease in correlation with disease severity (increasing Hoehn–Yahr scores). Demirci et al. (24) found corneal thickness to be significantly correlated with TBUT, blinking rates, and Schirmer test scores in PD patients.

Tamer et al. (18) measured the tear meniscus height in PD patients and controls and found abnormal tear meniscus height in 67.9% of the PD patients recruited in the study. Tear meniscus height was not linearly associated with the disease stage.

The use of optical coherence tomography to measure the thickness of corneal sublayers and tear meniscus proved to be a reliable method in evaluating the presence of dry eyes in Parkinson's disease patients.

#### Evaluation of blink rates

Blinking is necessary to maintain a healthy and regular tear film. Reduced blinking causes increased evaporation of aqueous components, resulting in subsequent contamination of the mucin layer and thinning of the tear film (34). Blinks are not only reduced but also are less effective with a decrease in amplitude and velocity in PD patients (35, 36). Due to impaired blinking, PD patients are at increased risk for dry eye. Inflammation, impairment of blinking, corneal sensitivity, and decreased tear secretion aggravate dry eye symptoms in PD (37).

PD patients were found to have significantly decreased blinking rates (BRs) compared to healthy controls (1, 18, 24, 27).

Tamer et al. (18) found blinking rates to be inversely correlated with total abnormal tear tests and with disease severity (H-Y scores). This is in concordance with other studies that also found a significant negative correlation between blink rates and H-Y scores in PD patients (24, 25, 32).

Fitzpatrick et al. (38) found significantly decreased blink rates in PD patients compared to healthy individuals during different everyday tasks such as reading a book or watching a video. They found no correlation between BR and disease severity, duration, or treatment.

Decreased blink rates, therefore, could be an important indicator that further tear tests are needed in order to properly diagnose dry eye syndrome in PD patients at risk.

### Dry eye staging

Based on the presence of symptoms and signs, dry eye can be classified into three grades of severity: grade 1 or mild, grade 2 or moderate, and grade 3 or severe (Table 2) (39).

**Table 2:** Dry eye classification of severity.

	**Grade 1 (mild)**	**Grade 2 (moderate)**	**Grade 3 (severe)**
Symptoms	+	++	+++
Signs	–	+ (reversible)	+ (irreversible → complications)

Symptoms: itching, dryness sensation, photophobia, ocular tiredness, and blurry vision.

Reversible signs: epithelial erosion, keratophakia filamentosa and punctata, short BUT, marginal blepharitis, and hyperemia.

Complications: corneal ulcers and neovascularization, keratinization, leukomas, retraction of conjunctival folds, and squamous epithelial metaplasia (permanent sequelae leading to reduced visual acuity).

## Impact on the quality of life

In Parkinson's disease patients, both motor and non-motor symptoms (including ophthalmological problems) contribute significantly to a decreased quality of life (QoL). PD patients with dry eye experience several symptoms that further worsen QoL. These symptoms include dryness, itching, redness, ocular fatigue and pain, excessive tearing, and decreased visual acuity. The presence of ocular discomfort due to dry eye was associated with greater interference with activities of daily living and with higher scores on the OSDI (40).

In a study by Borm et al. (41), 53% of PD patients reported that the presence of ophthalmologic symptoms had a moderate-to-severe effect on their quality of life, compared with 16% of controls. The greatest interference was experienced while reading, driving a car, watching television, and working on a computer. In another study, Borm et al. (42) measured the impact on daily life using the VFQ-25 (Visual Functioning-25 questionnaire). In total, 44% of PD participants reported poor QoL due to the presence of relevant ophthalmological disturbances. The severity of visual disturbances is also correlated with an increased risk for falls as PD patients compensate for their motor and postural impairments with visual guidance (43, 44).

In patients suffering from dry eye, the prevalence of depression and anxiety is approximately three times higher than that in patients without dry eye disease (45). This is especially important because depression and anxiety are among the most frequently reported neuropsychiatric disturbances in PD with a prevalence of up to 90% (46). Thus, patients with dry eye and Parkinson's disease are at increased risk for depression and anxiety. Treatment of dry eye with over-the-counter lubricants of the ocular surface improved patient-reported satisfaction levels and QoL to as high as 75% for patients with mild symptoms and 65% for patients with severe symptoms (47). However, PD-related motor impairments might interfere with the self-administration of ocular products, with PD patients experiencing limited independence from being unable to handle eye drop instillation themselves, thus further decreasing their quality of life (48).

## Management of dry eye in PD

Dry eye management usually begins with conventional over-the-counter ocular surface lubricants in the early stages and can progress to advanced therapies in more severe cases. In some of the cases, new therapies may be added to previous ones to increase the efficacy of the treatment. Treatment options are summarized in Table 3 (6, 49). However, symptomatic treatment of dry eye has not yet been studied, specifically for Parkinson's disease patients.

**Table 3:** Summary of treatment options for different stages of dry eye.

**Early stage**	**Moderate stage**	**Severe stage**
• Patient education • Elimination of environmental factors (e.g., air pollutants, hot and cold temperatures, and alcohol) • Elimination of precipitating medications (diuretics, beta-blockers, antihistamines, tricyclic antidepressants, antipsychotics, and antiparkinsonian drugs) • Artificial tear substitutes (cellulose ethers, carbomers, polyvinyl alcohol, sodium hyaluronate, and povidone) • Eyelid therapy (warm compresses and eyelid hygiene) • Correction of eyelid abnormalities (if present) • Treatment of contributing factors (e.g., blepharitis) • Treatment of underlying systemic disease	Early-stage treatment• + • Anti-inflammatory agents (topical steroids or cyclosporin) • Supplementation with omega-3 fatty acids • Punctal plugs • Moisture chamber glasses	Moderate-stage treatment• + • Oral anti-inflammatory agents (short-term corticosteroids or tetracycline) • Mucolytic agents • Autologous serum tears • Therapeutic contact lenses • Permanent punctal occlusion • Surgical intervention (tarsorrhaphy)

### Patient education

Patient education is an important step in the care management of people with chronic illnesses such as Parkinson's disease because it provides support and information to patients and caregivers while also improving self-care, treatment compliance, patient wellness, and physical function through exercise. Many patient education programs have been developed worldwide with various improvements in QoL in PD patients (50–54).

**Figure 3:**
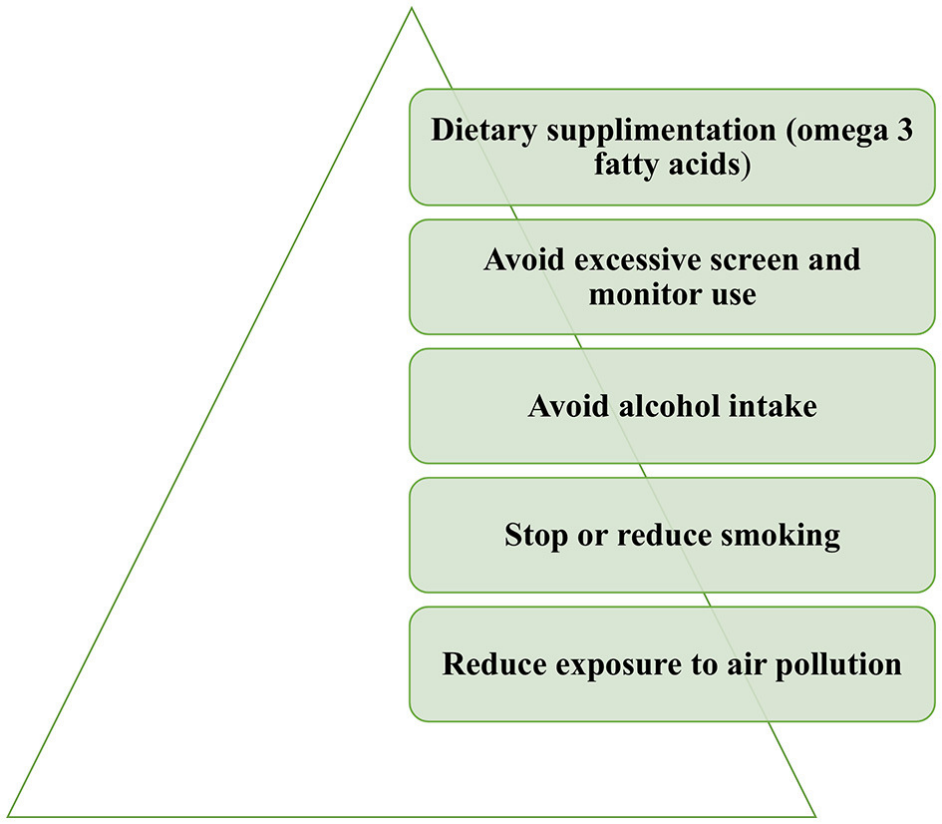
Lifestyle changes recommended for dry eye management.

Proper patient education is also essential for dry eye. The implementation of certain lifestyle and behavioral changes could alleviate dry eye symptoms (Figure 3). Exposure to air pollution or other environmental irritants, including tobacco smoke, should be limited. Cigarette smoking was found to have adverse effects on tear protein and on the lipid layer of the tear film and is associated with dry eye (55, 56). Excessive monitor usage should also be avoided. Dietary changes should be implemented with the consumption of omega-3 fatty acids and the limitation of alcohol intake (57).

### Elimination of precipitating medications

Antiparkinsonian medications such as levodopa, benzhexol, or pramipexole are known determinants of dry eye. Other implicated medications are also antipsychotics and antidepressants, as depression and psychosis are two major neuropsychiatric comorbidities in PD patients.

While the elimination of precipitating medications is recommended, that is not always the case for Parkinson's disease patients. Antiparkinsonian medication is crucial for the improvement of PD for both motor and non-motor symptoms. Clinicians should consider, if possible, switching between classes of antiparkinsonian medications if a certain treatment is considered to be the cause of dry eye symptoms. For example, amantadine was found to induce corneal endothelial toxicity in a dose-dependent manner (58), thus potentially being involved in treatment-induced dry eye.

On the other hand, levodopa replacement therapy has been shown to improve the blinking rates of PD patients (35). As discussed above, blinking is involved in the pathogenesis of dry eye, thus, an improvement in blinking might alleviate dry eye symptoms for these patients. The idea that patients should consciously increase their blink rates is difficult to achieve in the case of Parkinson's disease.

### Artificial tear substitutes

Artificial tear substitutes are inorganic solutions containing electrolytes, surfactants, and viscosity agents that aim to lower the surface tension of the tear film, enhance tear volume by forming a hydrophilic layer on the ocular surface, and prevent bacterial growth, thus reducing the symptoms of dry eye (59). Cellulose ethers, carbomers, polyvinyl alcohol, sodium hyaluronate, or povidone are the main components of most artificial tear substitutes. Each tear substitute has its own properties; hence, treatment should be individualized according to each patient's deficit. In the general population, substitute treatment with added lubricants and osmoprotectants has been shown to increase patient satisfaction levels over a short period of time (60, 61). However, this may not be the case for Parkinson's disease patients. The application of artificial tear substitutes requires increased manual dexterity. As PD patients struggle with fine-motor dexterity tasks due to motor impairments, treatment adherence is expected to be low. It is well-known that treatment burden is a serious issue for both PD patients and their caregivers, causing poor adherence to treatment, poor quality of life, and poor health outcomes (62).

### Advanced-stage therapies

In cases where artificial tear substitute treatment is insufficient, topical anti-inflammatory treatment may be efficient. Topical cyclosporine proved to have a high success rate for patients with mild-to-severe dry eye disease (63) and seemed to prevent the progression of dry eye symptoms over a period of 12 months (64). Topical corticosteroids have been reported to reduce corneal fluorescein staining and improve ocular irritation (65). Patients should be monitored, as prolonged treatment with corticosteroids may cause cataract formation and increased intraocular pressure.

In ocular surface diseases, obstruction of the lacrimal drainage to preserve the tears on the ocular surface can be achieved with punctal plugs, which are biocompatible silicone devices. The use of punctal plugs has been shown to decrease the use of tear substitutes and improve symptoms in dry eye patients (66). Complications of punctal plug use include partial migration or extrusion, which can cause local irritation or even canaliculitis and keratitis, loss, epiphora, punctal stenosis, and infectious complications (pyogenic granuloma) (67). Permanent punctal occlusion by laser or thermal cauterization can be beneficial in severe cases. An alternative for permanent punctal occlusion may be labial mucous membrane grafting, especially in patients with conjunctival cicatricial changes (68).

Moisture chamber glasses or spectacles (MCSs) are prosthetic devices that provide a comfortable and moister ocular environment by preventing the evaporation of tears and protecting the eyes from irritants such as wind, dust, or pollen. In a study by Shen et al. (69), significant improvements in ocular comfort and ocular parameters, tear meniscus height (TMH), non-invasive tear break-up time (NI-BUT), and tear film lipid layer thickness were found in the MCS group (dry eye subjects who wore MCSs for a period of 90 min) compared to the control group. MCSs are a feasible, non-invasive, alternative treatment for dry eye, especially for patients exposed to harsh environmental conditions.

The temporal or permanent closure of the eyelids (tarsorrhaphy) can be used in severe, refractory cases of dry eye. It allows a better distribution of the tear film on the surface of the eyes by decreasing the rate of evaporation of the tear film (70). It was proven to be very effective in the management of ocular surface problems, including dry eye, with a success rate as high as 90% and minor complications (71).

These treatments have not been specifically studied for Parkinson's disease patients.

## Sjögren's syndrome and Parkinson's disease

Sjögren's syndrome (SS) is an autoimmune disorder characterized by the presence of dry mouth, dry eyes, and recurrent episodes of salivary gland enlargement due to keratoconjunctivitis sicca and focal lymphocytic sialadenitis (72). It has been associated with central nervous system abnormalities such as seizures, cognitive dysfunction, aseptic meningoencephalitis, focal cerebral deficits, multiple sclerosis-like symptoms, and movement disorders (73).

The risk of Parkinson's disease was found to be 1.37 times greater in patients with autoimmune rheumatic diseases than in controls in a nationwide population-based cohort study (74). Furthermore, the incidence of PD was higher in SS patients (2.5%; 215 out of 8,422 patients); thus, primary and secondary SS patients were considered to have a higher risk of developing Parkinson's disease (74). The pathogenesis of this phenomenon is unclear. It is thought to be due to an autoimmune process aimed against the basal ganglia that could involve anti-SSA and SSB antibodies or anti–beta2-glycoprotein IgG antibodies (75, 76).

Several cases of SS associated with Parkinsonism have been described in the literature (75, 77–79). In most cases, antiparkinsonian drugs did not improve the neurological signs and symptoms, while corticosteroid treatment variably improved the symptomatology in some cases.

A diagnosis of Sjögren's syndrome should be considered in patients with Parkinsonian features complaining of xerostomia and dry eye.

We recommend that dry eye assessment, a critical element of vision considered vital in Parkinson's disease, be added to the recently described dashboard system for the vitals of PD patients (80).

## Conclusion

In Parkinson's disease patients' dry eye is a frequent complaint and has a negative impact on their health-related quality of life. Reduced blink rates due to dopamine depletion in the CNS, the presence of Lewy bodies in the substantia nigra, sympathetic and parasympathetic ganglia with subsequent autonomic system dysfunction, changes in meibum lipid composition and tear proteins, and changes in PD medications all play a role in the complex pathogenesis of this disorder. Various treatments for dry eye are available, but most of them, if not all, have not been specifically studied for Parkinson's disease patients. Instillation of artificial tear substitutes and removal of incriminated medications may not be feasible in PD patients. The care of these patients should always include an ophthalmologist as part of a multidisciplinary team. More studies are needed to explore this heterogenous syndrome in PD.

## Author contributions

LU, SD, and CF-P worked on the conception and design of the article. LU carried out the search and drafted the article. KRC, CF-P, and SD revised the article for important intellectual content. All authors contributed to the article and approved the submitted version.
